# Long-term use of a neural prosthesis in progressive paralysis

**DOI:** 10.1038/s41598-018-35211-y

**Published:** 2018-11-14

**Authors:** Yoji Okahara, Kouji Takano, Masahiro Nagao, Kiyohiko Kondo, Yasuo Iwadate, Niels Birbaumer, Kenji Kansaku

**Affiliations:** 1 Systems Neuroscience Section, Department of Rehabilitation for Brain Functions, Research Institute of National Rehabilitation for Persons with Disabilities, Saitama, Japan; 2 Department of Neurological Surgery, Chiba University Graduate School of Medicine, Chiba, Japan; 3 Department of Neurology, Tokyo Metropolitan Neurological Hospital, Tokyo, Japan; 4 Department of Neurology, Yoka Hospital, Hyogo, Japan; 5 Institute for Medical Psychology and Behavioural Neurobiology, University Tübingen, Tübingen, Germany; 6 Wyss Center for Bio and Neuroengeneering, Geneva, Switzerland; 7 Department of Physiology and Biological Information, Dokkyo Medical University School of Medicine, Tochigi, Japan; 8 Brain Science Inspired Life Support Research Center, The University of Electro-Communications, Tokyo, Japan

## Abstract

Brain–computer interfaces (BCIs) enable communication with others and allow machines or computers to be controlled in the absence of motor activity. Clinical studies evaluating neural prostheses in amyotrophic lateral sclerosis (ALS) patients have been performed; however, to date, no study has reported that ALS patients who progressed from locked-in syndrome (LIS), which has very limited voluntary movement, to a completely locked-in state (CLIS), characterized by complete loss of voluntary movements, were able to continue controlling neural prostheses. To clarify this, we used a BCI system to evaluate three late-stage ALS patients over 27 months. We employed steady-state visual evoked brain potentials elicited by flickering green and blue light-emitting diodes to control the BCI system. All participants reliably controlled the system throughout the entire period (median accuracy: 83.3%). One patient who progressed to CLIS was able to continue operating the system with high accuracy. Furthermore, this patient successfully used the system to respond to yes/no questions. Thus, this CLIS patient was able to operate a neuroprosthetic device, suggesting that the BCI system confers advantages for patients with severe paralysis, including those exhibiting complete loss of muscle movement.

## Introduction

Brain−computer interface (BCI) technology is employed as neural prostheses for communication and control^1,2^. The technology uses brain signals from electrodes or sensors placed either outside or inside the scalp. BCI techniques are expected to improve the quality of life of paralyzed individuals, being of particular benefit to those lacking voluntary muscle control.

Amyotrophic lateral sclerosis (ALS) is a neurodegenerative disease associated with progressive impairment of motor functions, eventually leading to a locked-in syndrome/state (LIS)^3^ characterized by very limited voluntary movement, and finally leading to a completely locked-in state (CLIS) characterized by complete loss of voluntary movement^4,5^. Many clinical studies on BCI-based communication in ALS patients have been performed^6–10^. Gilija *et al*. measured neural activities in the motor cortices of two ALS patients for 1 year after electrode implantation and found that both patients could readily manipulate cursors controlling computer screens^11^. Vansteensel *et al*. reported the successful use of a fully implanted BCI to read the electrocorticography (ECoG) signals of an ALS patient with LIS^12^. However, it remains unclear whether ALS patients who progress from LIS to CLIS can continue to control neural prostheses using neuroelectric activity. The only study in which an ALS patient progressed from LIS to CLIS using an invasive ECoG driven BCI could not achieve reliable communication with the patient after entering CLIS^13^. Chaudhary *et al*. recently found that none of four CLIS patients could communicate significantly over extended periods of time using electroencephalography (EEG)/frequency classification devices but could give yes/no answers to short questions employing functional near-infrared spectroscopy (fNIRS) communication^14^.

Here, we applied our in-house BCI system; we used steady-state visually evoked potentials (SSVEPs)^15^ of scalp EEG to allow three late-stage ALS patients to communicate over a period of 27 months. One of these patients progressed to CLIS but continued to operate the system accurately. To this end, in contrast to a previous study^14^, we demanded only covert attention shifts to flickering light sources; the CLIS patient obviously retained some visual function with light–dark discrimination.

## Results

**Figure 1:**
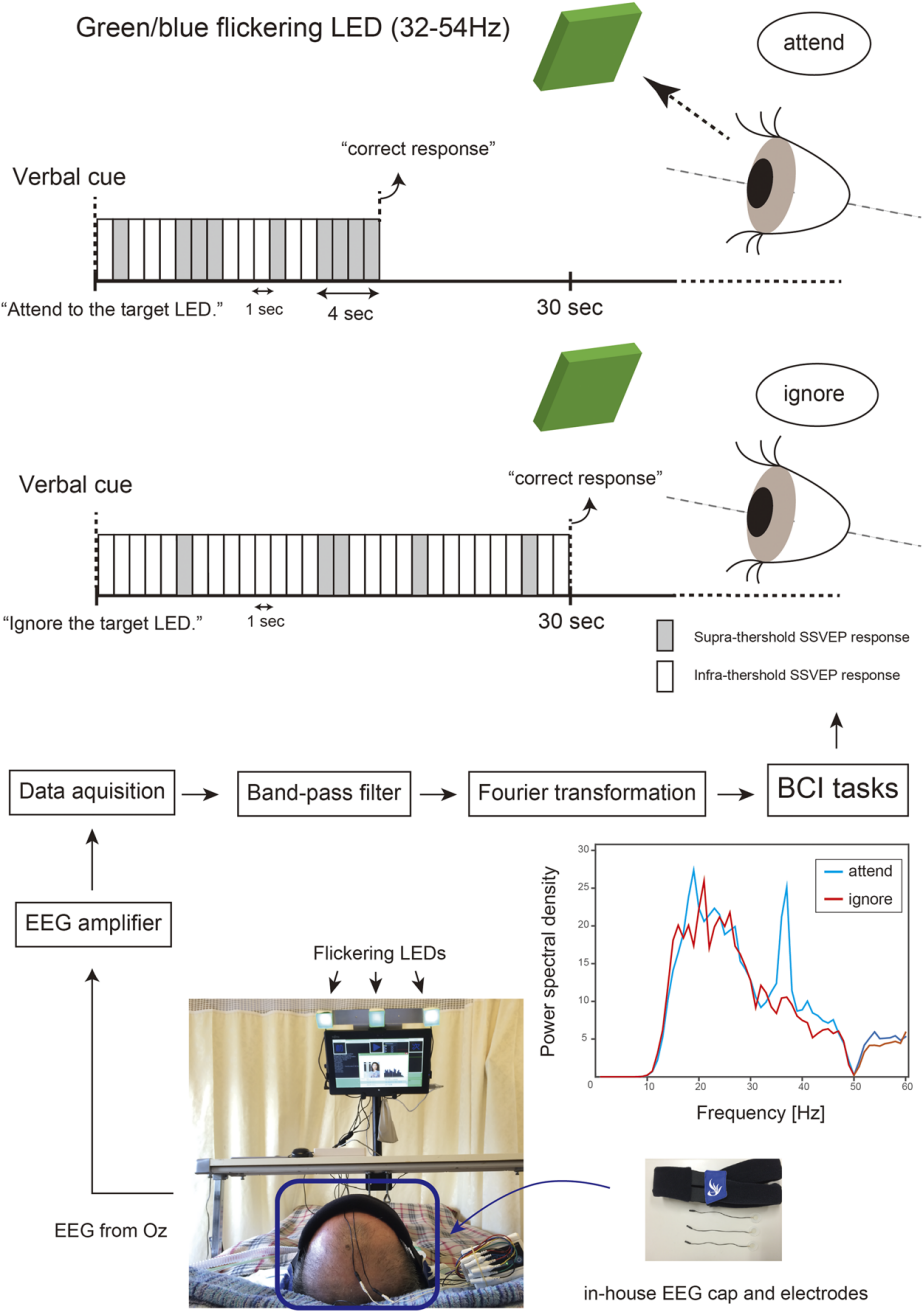
Experimental setting. SSVEP responses were recorded from a single electrode at Oz on each participant’s scalp. All participants laid on a bed, and each flickering LED was placed 60 cm from the participants’ eyes. LED frequencies of 32–54 Hz were applied. Before starting a session, we determined the PSD threshold and LED frequency. In each trial, following verbal instructions, the participants were required to attend to or ignore the flickering target LED for 30 seconds (attend/ignore task). The target LED was randomly assigned by the experimenters in each trial. The participants were instructed as follows: “Attend to the target LED” or “Ignore the target LED”. When the participants were asked to attend to the target LED, and the PSD exceeded the threshold for 4 seconds, we classified the response as a correct answer. When the instruction was to ignore the target LED, if the PSD remained below the threshold for 30 seconds, the response was classified as a correct answer. The participants completed the attend/ignore task trial 6 or 12 times in one session.

We used a bedside BCI system to analyze SSVEPs recorded over the visual cortex while the participant focused his/her visual attention on flickering stimuli^15^. Following verbal instruction, each patient was required to attend to, or ignore, a flickering light-emitting diode (LED) for 30 seconds per task trial. Online performance was evaluated by calculating a power spectral density (PSD) value from the measured SSVEP signals (Fig. 1).

**Figure 2:**
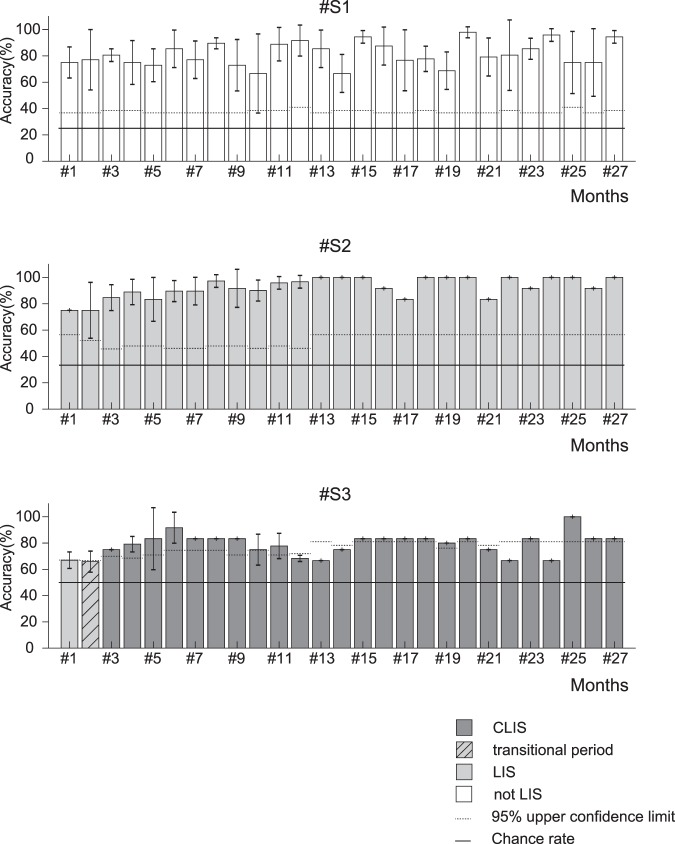
Performance of amyotrophic lateral sclerosis (ALS) patients #S1, #S2, and #S3 on the EEG-BCI system attend/ignore task. In the attend/ignore task, online accuracy was averaged for each month in all participants. The mean online performance in the attend/ignore task over all experiments and patients (191 sessions and 2,052 task trials) was 84.3%, and the means for the individual participants were 81.3% (#S1), 92.5% (#S2), 78.9% (#S3). The black broken horizontal lines indicate the upper confidence limits^16^ for results obtained by chance during each month; we calculated the proportions of results below the 95% confidence interval. The black solid horizontal lines indicate the chance rate of each class (#S1: four-class, 25%, #S2: three-class, 33.3%, #S3: two-class, 50%). The light gray bars indicate that the patients had LIS. The light gray bars with slanted stripes indicate that the patient was transitioning from LIS to CLIS. Note that patient #S3 showed BCI performance with reliable accuracy even after progression to CLIS.

The means overall online performance for each individual were 81.3% for #S1 (theoretical chance rate: 25.0%), 92.5% for #S2 (theoretical chance rate: 33.3%), and 78.9% for #S3 (theoretical chance rate: 50.0%) (Fig. 2). Overall, the accuracy rates exceeded the upper confidence limits^16^ throughout the experiments (we applied the 95% confidence limits for a chance result; #S1: 27.5%, #S2: 37.0%, and #S3: 55.8%). The accuracies were significantly higher than the upper confidence limits over the entire 27 months (paired t-test, #S1: p = 0.48 × 10^−21^, #S2: p = 0.50 × 10^−23^, and #S3: p = 0.58 × 10^−13^), and the median over all online performance was 83.3%. In terms of the monthly confidence limits, #S1 and #S2 had accuracies higher than these limits during all months, and #S3 attained accuracies above the confidence limits during 18 of the 27 months and in 27 of 40 sessions (see Supplementary Tables 1 and 2 for details).

When accuracies were evaluated every 9 months, the mean accuracies of the three patients gradually increased (81.6%, 84.0%, and 86.7%). For each participant, we compared the accuracies over time using one-way repeated-measures ANOVA. The main effect of period was significant in patient #S2 (p = 0.02). Also, significant differences according to the post-hoc Tukey–Kramer test (p = 0.04) were observed for all patients between the first and last 9 months.

**Figure 3:**
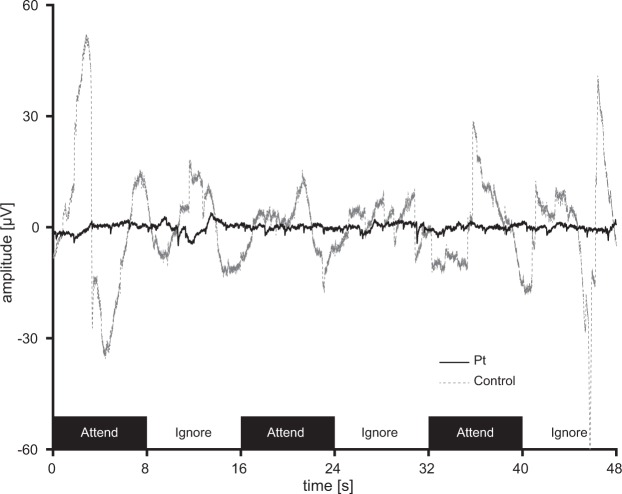
Electrooculogram acquisition in a normal volunteer and patient #S3. We recorded an electrooculogram (EOG) during BCI operation. The black bars indicate the times at which we instructed the subject to attend to the LED. The EOG of patient #S3 is shown as a solid black line. Note that the amplitudes were <3 μV and showed no systematic association with task timing. The gray dotted line is a representative EOG from an able-bodied volunteer operating the same BCI system via covert attention; this EOG exhibits large peaks of a few ten μV.

In patient #S3, electrooculography (EOG) performed during BCI operation was associated with amplitudes <3 μV and lacked any systematic association with task timing (Fig. 3; 20 μV corresponded to a visual angle of approximately 1° in our setting). We found no significant differences in EOG amplitude between attend and ignore status in either patient #S3 or a healthy volunteer (p = 0.59 and p = 0.31 for the patient and volunteer, respectively; two-tailed t-test). Patient S3 (with CLIS) controlled the BCI using covert attention, thus without directly gazing at the target LED^3,17–20^.

In addition to attend/ignore tasks, we also performed *ad hoc* testing; patients #S1 and #S2 were asked to use the BCI system to operate in-house audio software on demand. Patient #S1 employed three LEDs to this end, corresponding to “Play”, “Next track”, and “Previous track”. Patient #S2 used two LEDs, corresponding to “Play next track” and “Play previous track”. These trials were conducted on most experimental days to improve BCI performance with training; both patients were able to operate the audio player on demand with good scores by modified QUEST 2.0 (Quebec User Evaluation of Satisfaction with assistive Technology) (S1: 25/30, S2: 25/30)^21^ and we refrained from asking BCI related questions because of the instability of the health condition in patients.

We also prepared patient #S3 (with CLIS) for future use of the BCI in daily life. We used the attend/ignore task to answer yes/no questions posed by family members. The results suggested that the responses might not be reliable; therefore, to avoid false-positive results, we occasionally used a yes/no method consisting of several successive attend/ignore trials. For example, when a family member requested that the patient choose a Father’s Day present, we employed a yes/no method consisting of nine attend/ignore trials. One question was “For your father, do you want to give your first-choice present (a beer gift box)? If you do, please attend to the LED”. We posed nine similar questions. The BCI response revealed nine consistent answers; thus, a particular present was successfully chosen (see Supplementary Fig. 1 for details).

**Figure 4:**
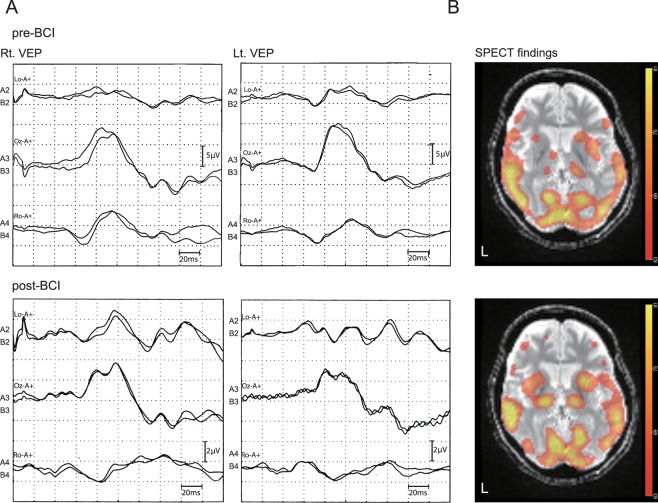
Physiological and radiological examinations in patient #S3. (**A**) VEP findings pre-BCI and 6 months post-BCI. Flash visually evoked potentials (VEPs) were recorded. A reference electrode was placed on Fz, and a ground electrode was positioned on the earlobe. VEPs were observed before and after the BCI intervention. (**B**) SPECT findings pre-BCI and 6 months post-BCI. ^99m^Tc-ethyl cysteinate dimer (ECD)-SPECT images were acquired. These images were transformed to a normalized brain (http://imaging.mrc-cbu.cam.ac.uk/imaging/MniTlairach) and were overlaid on a T2-weighted image of the patient. Note that an increased Z-score value at the left thalamus was found from pre-BCI to 6 months post-BCI (pre: − 1.0985, 6 months: − 0.7775).

Upon clinical examination of #S3, VEPs were observed both before and after BCI intervention (Fig. 4A). SPECT showed that, despite broadly decreased regional blood flow in the fronto-temporal lobes, the Z-score for regional blood flow of the left thalamus increased from before to 6 months after BCI intervention (pre: −1.0985; 6 months: −0.7775) (Fig. 4B). Her accuracy in terms of BCI system control increased over the first 6 months (Fig. 2). Unfortunately, she died of sudden cardiac arrhythmia, possibly caused by brainstem atrophy, just 9 days after successful use of the BCI system.

## Discussion

We recorded EEG-SSVEP signals in three late-stage ALS patients operating a BCI system over a period of 27 months. The patients showed sustained accuracy of control and voluntarily manipulated the amplitude to achieve task performance accuracies >80%, and the performance increased even with progression of the disease. Furthermore, we observed for the first time that an ALS patient who progressed from LIS to CLIS continued to operate the visual EEG/BCI system with high accuracy.

Although previous studies have suggested that visual EEG-based BCI systems might be difficult or impossible to use in late-stage ALS^5^, due to the loss of clear vision induced by drying of the corneas or corneal ulcers^9,13^, in our study, patients #S1, #S2, and #S3 had no such problems, probably due to the meticulous care provided by dedicated caregivers. This is consistent with a prior study showing preservation of the visual pathways in CLIS patients undergoing corneal care^22^ despite absence of voluntary eye movements.

We evaluated long-term, non-invasive EEG-BCI use in patients #S2 (with LIS) and #S3 (with CLIS development after LIS). Non-invasive BCI applications for LIS patients have already been reported^5,23,24^. In one of those reports, a patient with LIS caused by a brainstem stroke used a visually based P300 BCI system for 13 months^25^. In previous reports that explored how to communicate with patients who develop CLIS after LIS, an auditory-based P300 BCI system combined with epidural ECoG was used, but no communication was possible once the patients progressed to CLIS^8,9^. Three studies on CLIS patients used fNIRS to detect differential brain responses to simple questions^10,14,26^, although the technique remains controversial^27^.

Kübler and Birbaumer, in a meta-analysis of all relevant controlled single trials, concluded that EEG-based BCIs do not yield interpretable communication in CLIS patients^5^. Our data from patient #S3 shows otherwise. There are several possible reasons for this, including the present patients had intact vision because of extensive treatment of their eye conditions, and they were able to perceive at least flickering light and thus the SSVEP recordings. None of the CLIS patients evaluated by Kübler and Birbaumer used SSVEP-based BCI, which may require less attentional resources compared with other BCI systems featuring P300 spellers^25^, neurofeedback-acquired slow cortical potentials^28^, or self-control of sensorimotor rhythm via imagery^2^. The task of the BCI system by Chaudhary *et al*., answering mentally yes or no to many questions within a 30 sec period, may have been too attention demanding for CLIS patients to achieve reliable and high performance. In addition, for an EEG based BCI system an answering period of 30 sec while appropriate for the slow changing blood oxygenation of NIRS may be far too long to find the appropriate time interval of the mental answer within those 30 secs. The larger variability of EEG oscillations allows many more feature selections for an AI classifier than the NIRS-BCI system used by Chaudhary *et al*.^14^ and thus does not need such a long answering period. However, even in this SSVEP-BCI despite the more or less automatic elicitation of the brain response, the task is attention demanding but may allow faster automatization of the cognitive response.

The cognitive processes underlying attention and motivation may deteriorate with long-term, complete paralysis in ALS patients^5,29^, such that early intervention with a BCI system after a patient acquires CLIS status may be important. Furthermore, the SPECT data we observed in the left thalamus (Fig. 4B) suggest that the BCI-based communication may have had a compensatory effect in terms of brain reorganization and rehabilitation, despite there being some neuronal loss in the thalamus during late-stage ALS^30^.

In this study, we performed a longitudinal evaluation of a BCI system using SSVEP signals in three late-stage ALS patients, and showed increasing and sustained accuracy of control. Patient #S3, who progressed to CLIS, was able to continue operating the system. Indeed, she used the BCI system to respond to yes-no questions when family members made a request, suggesting that this methodology has the potential to improve and maintain the quality of life of patients with complete paresis.

## Methods

Three ALS patients (#S1, #S2, #S3; two males; 64, 50, 37 years of age, ALS functional rating scale-revised (ALSFRS-R) scores = 0) participated in this study (Table 1). No participant underwent prior BCI training. The inclusion criteria were ALSFRS-R scores = 0, and the absence of CLIS at commencement of the experiment. Patient #S1 was able to move his neck and eyes and to control his immediate environment via residual muscle contractions. Patient #S2 had been diagnosed with LIS 1 year before the experiment commenced. He could move his eyes and communicated using a transparent Kana board. Patient #S3 communicated using slight eye movements at the commencement of the study (e.g., a deviation to the right meant “yes”), but, 1.5 months after the study commenced, she lost all muscle movement. Two medical doctors independently diagnosed CLIS, where the doctors followed the criteria of Hayashi and Kato^4^. We used EOG to detect residual eye movement.

**Table 1:** Demographic and clinical characteristics of the ALS patients.

	Age	Sex	ALSFRS-R	Disease duration (years)	Tracheotomy	State	Number of LEDs
S1	64	Male	0	19	Yes	Residual muscle control	3
S2	50	Male	0	24	Yes	LIS	2
S3	37	Female	0	6	Yes	LIS to CLIS	1

ALSFRS-R, ALS Functional rating scale revised; LIS, locked-in state; CLIS, completely locked-in state.

The present study was approved by our institutional ethics committee at the National Rehabilitation Center for Persons with Disabilities. Written informed consent was obtained from all participants and their legal representatives. All experiments were carried out in accordance with the approved guidelines.

This study was conducted in a hospital (#S1) or at the patients’ homes (#S2 and #S3) over 27 months. We prepared a 4 × 4-cm LED flicker as a visual stimulus to elicit SSVEPs. The flickering LEDs were fixed 60 cm from the patients’ eyes. LED frequencies of 32–54 Hz^15^ were applied. The eyes of patient #S3 were usually slightly open, but she could not move her eyelids; thus, we opened her eyes wide using surgical tape. To avoid drying of their cornea, #S3 received eye drops and/or her eyes were closed passively, and #S1 and 2 blinked voluntarily.

One to four sessions were performed every month. Each session featured 6 or 12 task trials (attend/ignore tasks). A total of 191 sessions and 2,052 task trials were performed (98, 53, and 40 sessions and 1,176, 636, and 240 task trials for #S1, #S2, and #S3, respectively). The number of sessions varied depending on participants’ medical condition such as their fatigue, blood pressure level and respiratory condition. Before each session, to determine both a target EEG frequency and a threshold PSD obtained from the EEG data, we asked the participants to attend to the target LED three times, and to ignore it three times, for periods of 15 seconds each. During each task trial, participants were asked to follow verbal instructions to attend to or ignore the target LED randomly assigned by the experimenter for 30 seconds (Fig. 1).

When asked to attend, if the PSD of the frequency of the flickering LED crossed over the threshold for 4 seconds, the response was recognized as a correct answer. When asked to ignore, if the PSD was kept below the threshold for 30 seconds, the response was accepted as correct. Attending and ignoring tasks were alternately presented during each trial. Online feedback regarding the task performance was verbally delivered, and online accuracies were recorded.

Each patient required a different number of LEDs: three for #S1, two for #S2, and one for #S3. A reduction in the number of choices assists BCI use as disease severity increases^7^. Patient #S1 exhibited residual ocular movements, but #S2 sometimes lost gaze control. Thus, #S1 and #S2 attended to target LEDs both overtly and covertly. Because #S3 had lost residual ocular movements, she covertly attended to the target LED. For #S3, one LED was set approximately 15° from the center of the optic axis, allowing for easy attention shifting without gazing^17,18^. EOGs were recorded over several sessions to evaluate residual eye movements.

An in-house environmental control system was used that consisted of a tablet PC, an EEG amplifier (AvatarEEG; Electrical Geodesics, Inc., Eugene, OR), and 1–3 LED flickers with a peripheral interface controller.

The EEG data including SSVEPs were recorded using a single Oz channel. The electrode was referenced to Fpz and grounded to AFz. Electrodes were fixed using an in-house, non-drying solid gel with long-lasting stability^31^. The position of each electrode was determined using the 10–10 EEG coordinate system^32^. Each EEG was digitized at 500 Hz and stored.

The obtained signals were filtered with a 16-order 50 Hz notch filter and a 12-order 15 Hz high-pass filter. The filtered signals were buffered for 4 seconds, and a Fourier transformation was applied to the buffered signals. These processes were conducted every 1 s. Online performance was evaluated by calculating the PSD. Each PSD value for attention and ignoring was calculated as follows:

$$PS{D}_{ignore}(x)={|\sum _{k={t}_{i}(0)}^{{t}_{i}(4N-1)}f(x){e}^{-i(\frac{2\pi kx}{N})}|}^{2}$$

$$PS{D}_{attention}(x)={|\sum _{k={t}_{a}(0)}^{{t}_{a}(4N-1)}f(x){e}^{-i(\frac{2\pi kx}{N})}|}^{2},$$

where *PSD*_*attention*_*(x)* and *PSD*_*ignore*_*(x)* are power spectra obtained during attending to and ignoring the frequency *x. N* is the sampling rate. *ta(n)* and *ti(n)* denote the sample timing of the EEG signals for attention and ignoring. The frequency *x* was calculated as:

$${x}=fre{q}_{LED}+fre{q}_{offset},$$

where *freq*_*LED*_ and *freq*_*offset*_ are the frequency of the LED and the offset of frequency, respectively. *freq*_*offset*_ is a value ranging from −2 to 2, in increments of 0.5. The difference between *PSD*_*attention*_*(x)* and *PSD*_*ignore*_*(x)* was calculated for all *freq*_*offset*_, and the EEG frequency that provided the largest difference was selected as the target frequency. To determine the *freq*_*LED*_ for each participant, we performed several trials at different frequencies (27–54 Hz) before each session.

We determined a threshold for classification using the following formula:

$$Thre(x)=C\cdot (Diff(x))+\sqrt{PS{D}_{ignore}(x)}$$

$$Diff(x)=\,\sqrt{PS{D}_{attention}(x)}-\sqrt{PS{D}_{ignore}(x)},$$

where *Thre(x)* is a threshold of the target frequency and *C* is a constant value (0.6 ≤ C ≤ 0.8). C was determined according to the EEG amplitude of each participant; this reduced misclassifications in noisy environments (hospital or home). To determine C, we used the value of *Diff(x)* derived above. If *Diff(x)* was >2.0, C was set to 0.6; if *Diff(x)* was ≤2.0, C was set to 0.8. For example, the *Diff(x)* values for #S1, #S2, and #S3 were 3.91, 3.69, and 1.19 μV, respectively. Thus, the C values for #S1, #S2, and #S3 were set to 0.6, 0.6, and 0.8, respectively.

The results of online performance over the entire period of 27 months were further evaluated by calculating the accuracies at 9-month intervals. For each participant, we performed one-way repeated-measures analysis of variance^33^ to compare the accuracies over these periods. A *post hoc* analysis (Tukey–Kramer test) was performed for each result in the individual analysis.

For patient #S3, four EOG electrodes were used to document CLIS. Two electrodes were placed on the lateral side of both eyes, and the other two electrodes were placed above and below the right eye. The EOG and EEG data were simultaneously digitized with a frequency of 500 Hz. The digitized signals were filtered with a 6-order bandpass filter from 0.1 to 30 Hz. We tested our system in normal volunteers and found that 20 μV corresponded to approximately 1° of visual angle.

For #S3, flash VEPs were recorded using the Neuropack X1 (MEB-2300, Nihon Kohden, Tokyo, Japan). A reference electrode was placed on Fz, and a ground electrode was positioned on the earlobe.

SPECT brain images were also acquired twice for patient #S3 (before and 6 months after the beginning of the experiments). #S3 underwent dual-SPECT evaluation using a double-headed gamma camera (PRISM-AXIS, Philips, Amsterdam, The Netherlands). ^99m^Tc-ECD was intravenously injected and flushed at resting state. Each reconstructed slice was transformed to a normalized brain (http://imaging.mrc-cbu.cam.ac.uk/imaging/MniTlairach) and superimposed on a T2-weighted image using eZIS (Fujifilm RI Pharma Co., Ltd., Tokyo, Japan). The eZIS incorporates SPM2 (Wellcome Trust Center for Neuroimaging, London, UK) to calculate Z-score values, which were obtained by comparing our images with the implemented SPECT images of age-matched healthy volunteers (ages 30–39). The Z-scores in the region of interest (left thalamus) were evaluated using an in-house Matlab software.

## Electronic supplementary material

Supplementary Materials
