# Supplementary material for: Long-term use of a neural prosthesis in progressive paralysis
Source: Sci Rep. 2018 Nov 14;8:16787. doi: 10.1038/s41598-018-35211-y (PMC6235856; doi:10.1038/s41598-018-35211-y)
Supplement: Supplementary file 1 — Supplementary Materials [file 41598_2018_35211_MOESM1_ESM.doc]

**Supplementary Materials**

**Title**

Long-term use of a neural prosthesis in progressive paralysis

**Authors**

Yoji Okahara# ^a,b^, Kouji Takano# ^a^, Masahiro Nagao ^c^, Kiyohiko Kondo ^d^, Yasuo Iwadate ^b^, Niels Birbaumer ^e,f^, Kenji Kansaku* ^a,g,h^

**Author affiliations**

^a^ Systems Neuroscience Section, Department of Rehabilitation for Brain Functions, Research Institute of National Rehabilitation for Persons with Disabilities, Saitama, Japan

^b^ Department of Neurological Surgery, Chiba University Graduate School of Medicine, Chiba, Japan

^c^ Department of Neurology, Tokyo Metropolitan Neurological Hospital, Tokyo, Japan

^d^ Department of Neurology, Yoka Hospital, Hyogo, Japan

^e^ Institute for Medical Psychology and Behavioural Neurobiology, University Tübingen, Tübingen, Germany

^f^ Wyss Center for Bio and Neuroengeneering, Geneva, Switzerland

^g^ Department of Physiology and Biological Information, Dokkyo Medical University School of Medicine, Tochigi, Japan

^h^ Brain Science Inspired Life Support Research Center, The University of Electro-Communications, Tokyo, Japan

^#^These authors contributed equally to this work.

*Correspondence should be addressed to:

Kenji Kansaku, M.D., Ph.D.

kansakuk@dokkyomed.ac.jp

**Supplementary Table 1: Accuracies and 95% upper confidence limits for results obtained by chance during each month.**

|  |  | #S1 |  |  | #S2 |  |  | #S3 |  |
| --- | --- | --- | --- | --- | --- | --- | --- | --- | --- |
| Month | # of trials | Upper confidence limit [%] | Accuracy [%] | # of trials | Upper confidence limit [%] | Accuracy  [%] | # of trials | Upper confidence limit [%] | Accuracy  [%] |
| #1 | 48 | 37 | 75 | 12 | 56 | 75 | 29 | 67 | 67 |
| #2 | 48 | 37 | 77 | 16 | 54 | 75 | 30 | 67 | 66 |
| #3 | 36 | 38 | 81 | 52 | 46 | 85 | 20 | 70 | 75 |
| #4 | 36 | 38 | 75 | 36 | 48 | 89 | 24 | 69 | 79 |
| #5 | 48 | 37 | 73 | 36 | 48 | 83 | 18 | 71 | 83 |
| #6 | 48 | 37 | 85 | 48 | 46 | 90 | 12 | 74 | 92 |
| #7 | 48 | 37 | 77 | 48 | 46 | 90 | 12 | 74 | 83 |
| #8 | 48 | 37 | 90 | 36 | 48 | 97 | 12 | 74 | 83 |
| #9 | 48 | 37 | 73 | 36 | 48 | 92 | 18 | 71 | 83 |
| #10 | 36 | 38 | 67 | 48 | 46 | 90 | 12 | 74 | 75 |
| #11 | 36 | 38 | 89 | 36 | 48 | 96 | 18 | 71 | 78 |
| #12 | 24 | 41 | 92 | 48 | 46 | 97 | 16 | 72 | 68 |
| #13 | 48 | 37 | 85 | 12 | 56 | 100 | 6 | 81 | 67 |
| #14 | 36 | 38 | 67 | 12 | 56 | 100 | 8 | 78 | 75 |
| #15 | 36 | 38 | 94 | 12 | 56 | 100 | 6 | 81 | 83 |
| #16 | 48 | 37 | 88 | 12 | 56 | 92 | 6 | 81 | 83 |
| #17 | 48 | 37 | 77 | 12 | 56 | 83 | 6 | 81 | 83 |
| #18 | 36 | 38 | 78 | 12 | 56 | 100 | 6 | 81 | 83 |
| #19 | 48 | 37 | 69 | 12 | 56 | 100 | 10 | 76 | 80 |
| #20 | 48 | 37 | 98 | 12 | 56 | 100 | 6 | 81 | 83 |
| #21 | 48 | 37 | 79 | 12 | 56 | 83 | 8 | 78 | 75 |
| #22 | 36 | 38 | 81 | 12 | 56 | 100 | 6 | 81 | 67 |
| #23 | 48 | 37 | 85 | 12 | 56 | 92 | 6 | 81 | 83 |
| #24 | 48 | 37 | 96 | 12 | 56 | 100 | 6 | 81 | 67 |
| #25 | 24 | 41 | 75 | 12 | 56 | 100 | 6 | 81 | 100 |
| #26 | 48 | 37 | 75 | 12 | 56 | 92 | 6 | 81 | 83 |
| #27 | 36 | 38 | 94 | 12 | 56 | 100 | 6 | 81 | 83 |

**Supplementary Table 2: Number of sessions in which the results were above the chance 95% upper confidence limits for each participant.**

|  | # of sessions | | |  |
| --- | --- | --- | --- | --- |
|  | Total | Over the upper confidence limits | Below the upper confidence limits | Probability [%] |
| #S1 | 98 | 93 | 5 | 94.9 |
| #S2 | 53 | 53 | 0 | 100 |
| #S3 | 40 | 27 | 13 | 67.5 |

**Supplementary Figure 1**

**Supplementary Figure Legend**

**Figure 1: #S3 chose a Father’s Day present using the BCI system.** This Figure shows the answer tree used. #S3 employed an AAC device to communicate with her mother and prepare a gift list for her father. On an experimental day, we used the yes/no method (nine attention/ignore task trials) to choose a Father’s Day present. Question 1 (Q1) consisted of three task trials. The first question was “For your father, do you want to give your first-choice present (a beer gift box)? If yes, please pay attention to the LED”. In the second question, we assigned yes to inattention to the LED, and in the third question, we assigned yes to attention to the LED. Question 2 (Q2) was “For your father, do you want to select your second-choice present (an organic beef assortment box)?”, and question 3 (Q3) was “Do you really want to give your second-choice present?” Q2 and Q3 also consisted of three task trials. As shown by the red lines in the figure, the first choice was denied and the second choice confirmed. Throughout the BCI operation, all nine answers were consistent, and a particular present (an organic beef assortment box) was thus successfully chosen.
